# A new crystal form of *Aspergillus oryzae* catechol oxidase and evaluation of copper site structures in coupled binuclear copper enzymes

**DOI:** 10.1371/journal.pone.0196691

**Published:** 2018-05-01

**Authors:** Leena Penttinen, Chiara Rutanen, Markku Saloheimo, Kristiina Kruus, Juha Rouvinen, Nina Hakulinen

**Affiliations:** 1 Department of Chemistry, University of Eastern Finland Joensuu Campus, Joensuu, Finland; 2 VTT Technical Research Center of Finland Ltd., Espoo, Finland; Auburn University, UNITED STATES

## Abstract

Coupled binuclear copper (CBC) enzymes have a conserved type 3 copper site that binds molecular oxygen to oxidize various mono- and diphenolic compounds. In this study, we found a new crystal form of catechol oxidase from *Aspergillus oryzae* (*Ao*CO4) and solved two new structures from two different crystals at 1.8-Å and at 2.5-Å resolutions. These structures showed different copper site forms (*met/deoxy* and *deoxy*) and also differed from the copper site observed in the previously solved structure of *Ao*CO4. We also analysed the electron density maps of all of the 56 CBC enzyme structures available in the protein data bank (PDB) and found that many of the published structures have vague copper sites. Some of the copper sites were then re-refined to find a better fit to the observed electron density. General problems in the refinement of metalloproteins and metal centres are discussed.

## Introduction

**Fig 1:**
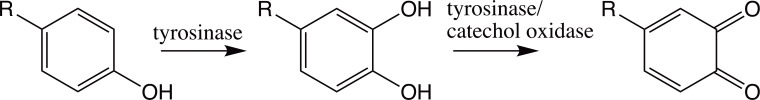
Tyrosinase and catechol oxidase activity.

Coupled binuclear copper (CBC) proteins contain a type-3 copper site that reversibly binds dioxygen [1]. The CBC protein family consists of oxygen carrier proteins haemocyanins, and various oxidative enzymes, including tyrosinases (EC 1.14.18.1), catechol oxidases (EC 1.10.3.1), and *o*-aminophenol oxidases (EC 1.10.3. 4) [2]. While the dioxygen binding capacity is a common feature of haemocyanins and enzymes in the CBC family, these enzymes often exhibit distinct substrate preferences for the catalysed oxidative reactions. Tyrosinase catalyses *o*-hydroxylation of monophenols to *o*-diphenols (mono-oxygenase activity) and the subsequent oxidation of *o*-diphenols to the corresponding *o*-quinones (diphenolase activity). Catechol oxidase only catalyses the latter reaction (Fig 1), and *o-*aminophenol oxidase catalyses oxidation of *o*-aminophenols to *o*-quinoneimines in the grixazone biosynthetic pathway [3]. All enzymes that belong to the CBC family utilize molecular oxygen, which is the final electron acceptor and is reduced to water. In addition to the above-mentioned CBC enzymes, a novel mono-oxygenase (called NspF) that can convert *o*-aminophenols to corresponding nitroso compounds has been recently found [4].

Many of the CBC proteins are structurally well characterized. The first crystal structure determined for CBC proteins dates back to year 1989, when the crystal structure of haemocyanin from *Panulirus interruptus* was deposited in the RCSB protein data bank (PDB) [5]. The first crystal structure of catechol oxidase from *Ipomoea batatas* was published in 1998 [6], and finally, the first crystal structure of tyrosinase from *Streptomyces castaneoglobisporus* was solved in 2006 [7]. In the last decade, more crystal structures have become available, and today, more than 70 structures (56 of those are tyrosinases or catechol oxidases) can be found in the PDB. However, no crystal structures of *o*-aminophenol oxidases or newly discovered novel mono-oxygenases are available. The molecular basis of different substrate specificities among the members of the family and reaction mechanism remain unclear.

Based on spectroscopic studies and more recent X-ray data, the active site of CBC enzymes has been described to exist at least in four possible forms: *oxy-*, *hydroperoxide*-, *met-*, and *deoxy*-forms [8]. In the *oxy*-form, the two Cu^II^ ions are bridged by a dioxygen molecule with a Cu-Cu distance of approximately 3.6 Å [9]. Oxyhaemocyanin exhibits an intense absorption band at 345 nm (ε = 20000 M^-1^cm^-1^), indicating the side-on peroxide bridging mode [10]. An absorption band at 340 nm is also observed for the *oxy*-form of tyrosinases and catechol oxidases [11–13]. In the *deoxy*-form, the pair of copper ions is reduced to Cu^I^ and the distance between them is increased to 4.6 Å [14]. Under aerobic conditions, the *deoxy*-form quickly binds dioxygen, leading to an active *oxy*-form [15]. A model is proposed for *met*-form that has two tetragonal Cu^II^ ions at a distance of 3.4 Å bridged by one endogenous ligand (such as hydroxide) [16–17]. The hydroperoxide binding mode of oxygen is observed in biomimetic studies of binuclear copper sites [18–19]. The characteristic absorption band of *μ*-1,1-hydroperoxide is suggested to appear at 400 nm [8]. It is discussed that the *hydroperoxide*-form could be a result of either oxygen binding to a reduced binuclear copper site or activation of peroxide for substrate hydroxylation [8].

The fungal catechol oxidase from *Aspergillus oryzae* (*Ao*CO4) has been heterologously produced in *Trichoderma reesei* [12], and the crystal structures of the full-length (4J3P) and a truncated form (4J3Q) of *Ao*CO4 have been solved at 2.5- and 2.9-Å resolutions, respectively [20]. In the full-length form, an unexpected copper site geometry with bound diatomic oxygen species has been observed. An oxygen atom, O2, of dioxygen species was found to be located 2.0–2.3 Å away from the copper ions, and an oxygen atom, O1, was located 2.6 Å away from the copper ions. The orientation of this diatomic oxygen species differs from other solved structures where the copper centre exists in *oxy*-form. In *oxy*-form, both oxygen atoms are located at equal distances from copper ions. The structure of *Ao*CO4 also raised a question concerning the molecular determinants of the substrate specificity between tyrosinases and catechol oxidases. The bulky phenylalanine residue Phe261 has been thought to prevent the access of monophenolic substrates to CuA to quench tyrosinase activity [7]. However, in the *Ao*CO4 structure, this phenylalanine is replaced by a valine residue, as typically found in tyrosinases. In addition, in *Ao*CO4, both copper ions are solvent exposed.

In the present study, a new crystal form of *Ao*CO4 that diffracted at a higher resolution was found. Two new crystal structures, called *met/deoxy* (an average of *met* -and *deoxy*-forms) and *deoxy*, were solved at 1.8- and 2.5-Å resolutions, respectively. The structures represented different coordination of the copper site compared with those in a previous study and demonstrated the ambiguity of structural interpretation due to rapid radiation damage that occurs during data collection from high-energy synchrotron sources. Copper ions may be fully or partially reduced during data collection, resulting in modifications in the active site. We also analysed and re-refined some of the copper sites in CBC enzymes to understand the dynamics at the binuclear copper site. Thorough analysis of the binuclear copper centre is a prerequisite to understand the molecular basis of tyrosinase- and catechol oxidase-catalysed reactions and associated dioxygen reduction to water.

## Results and discussion

### Overall structure

**Fig 2:**
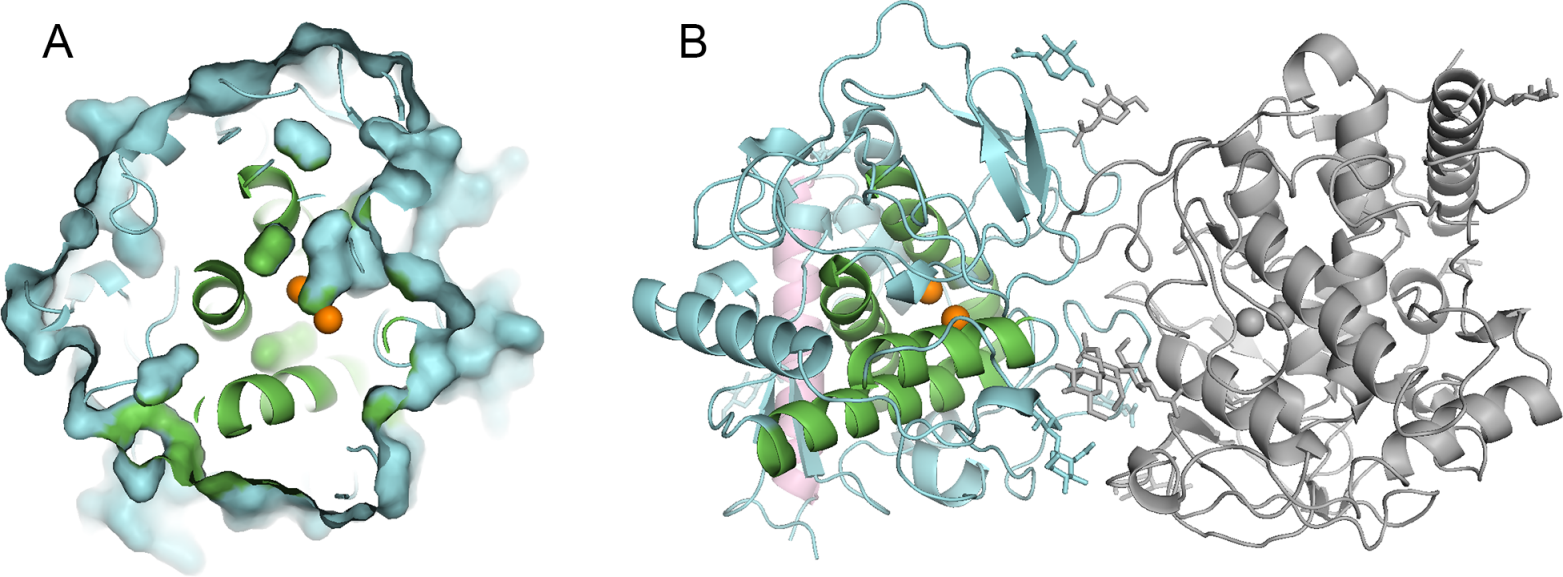
Overall structure of *Ao*CO4. **A** Molecular surface representation of a monomeric catechol oxidase from *Aspergillus oryzae* (in cyan) shows the accessibility of the binuclear copper centre to the protein surface. Copper ions are represented in orange. The central bundle of four α-helices is shown in green. **B** The dimeric structure of *Ao*CO4. The dimeric counterpart is in grey. The long N-terminal α-helix of the monomer is represented in pink. The observed carbohydrates are shown as stick models.

The overall structure of *Ao*CO4 shows a four-helix bundle around the copper-binding site, as seen in all CBC proteins (Fig 2). A long N-terminal α-helix, which is typical for the full-length form of *Ao*CO4, is observed in both structures. At the copper site, two T3 copper ions are each coordinated by three histidine residues. In *Ao*CO4, His102, His110, His119 and His284, His288 and His312 are responsible for CuA and CuB binding, respectively.

Two novel structures—i.e., *met/deoxy* (PDB code: 5OR3; 1.8 Å) and *deoxy* (PDB code: 5OR4; 2.5 Å)—can form a weak homodimer, as also earlier observed [20]. The new triclinic crystal form contains two homodimers in a crystallographic asymmetric unit. Based on PISA server analysis [21], the buried surface area in the dimeric subunits are 864 Å^2^ (molecules A and D) and 828 Å^2^ (molecules B and C) that indicates that *Ao*CO4 can exist as weak dimer in solution. Amount of dimer is proportional to the concentration of protein. In this triclinic crystal form, the citrate ion forms crystal contacts between molecules A and B, resulting in symmetry with two homodimers in the asymmetric unit (Figure A in S1 File). Our dynamic light scattering measurements also suggest that *Ao*CO4 can exist as a dimer in solution (Figure B in S1 File).

Both the *met/deoxy* and *deoxy* crystal structures show a similar glycosylation pattern to that previously reported for *Ao*CO4 [20]. However, N-acetylglucosamine (NAG) attached to Asn222 in molecule B and NAG attached to Asn30 in molecule C are not included in the final model of *met/deoxy* due to the observed very weak electron density. In the final model of *deoxy*, NAG attached to Asn222 in molecule A and mannose attached to Thr14 in molecule C are also excluded. Additionally, a mannose is attached to Thr5 in molecule A in the final model of *met/deoxy*. Otherwise, all of the same carbohydrate residues can be found in the triclinic crystal form, as observed in the trigonal crystal form. Glycans attached to Asn104, Asn222 and Asn348 expand the monomer-monomer interface area of the dimer, suggesting that the carbohydrates might be involved in dimerization.

### Copper site of *Ao*CO4 in the *met/deoxy* and *deoxy*-forms

**Fig 3:**
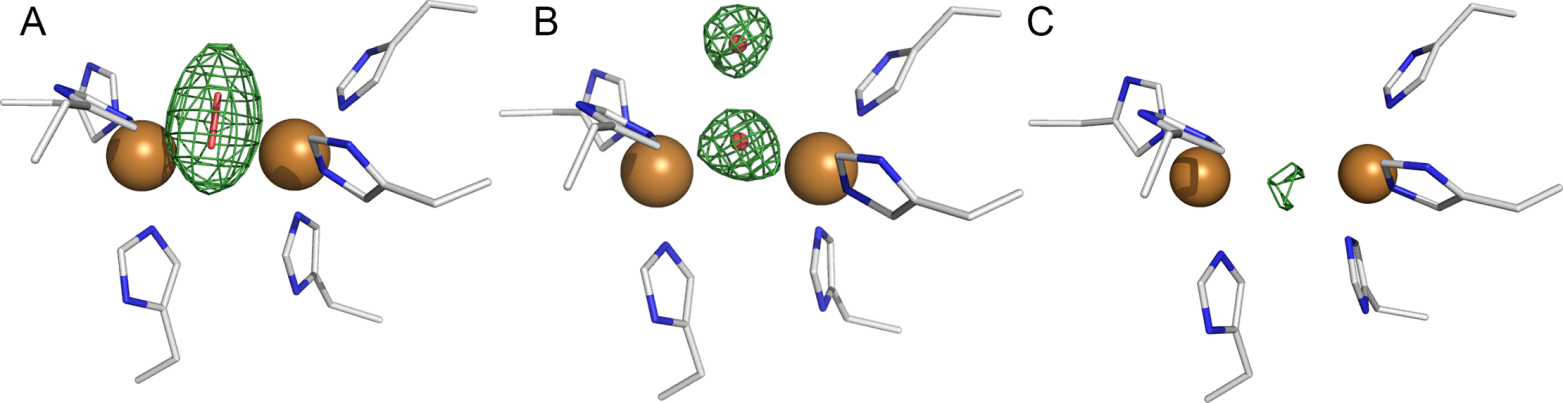
Copper sites in the crystal structures of catechol oxidase from *Aspergillus oryzae*. An *F*_*o*_*−F*_*c*_ omit map of oxygen species at the copper site is shown in green at the 3σ contour level. **A** 4J3P (now shown with a peroxide moiety) **B** *met/deoxy* (molecule B is shown) and **C** *deoxy* (molecule B is shown).

The two new crystal structures showed different forms of copper sites than our previously solved crystal structure of full-length *Ao*CO4 (4J3P), as shown in Fig 3. In the published 4J3P structure, a bound diatomic oxygen species is located between the two copper ions, with a Cu-O2 distance of 2.0–2.3 Å and Cu-O1 distance of 2.6 Å. The Cu-Cu distance was 4.2 Å. Here, the two new crystal structures do not contain this diatomic oxygen species and the Cu-Cu distances were increased from 4.2 Å in 4J3P to 4.3–4.7 Å in *met/deoxy* and *deoxy* structures. These structures were also different compared with each other. From the *met/deoxy* data at a 1.8-Å resolution (Fig 3B), a stronger electron density peak between the copper ions was observed than in the *deoxy* data at a 2.5-Å resolution (Fig 3C).

**Fig 4:**
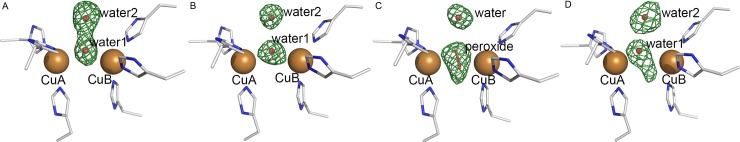
Copper site of the *met/deoxy* structure of catechol oxidase from *Aspergillus oryzae*. An *F*_*o*_*−F*_*c*_ omit map of oxygen species at the copper site is shown in green at the 3σ contour level. **A**: molecule A, the Cu-Cu distance was 4.3 Å, and the distance between water molecules was 2.5 Å. **B**: molecule B, Cu-Cu distance was 4.4 Å, and the distance between water molecules was 2.6 Å, **C**: molecule C, the Cu-Cu distance was 4.4 Å, and the distance from atom O2 of the peroxide moiety to water was 2.5 Å. **D**: molecule D, the Cu-Cu distance was 4.5 Å, and the distance between water molecules was 2.6 Å.

There were also interesting variations in the binuclear centre between the molecules in the asymmetric unit (Fig 4). In the *met/deoxy* data, the water1 molecule refined well with a distance of 2.3–2.4 Å from the copper ions in molecules A, B and D. An additional water2 molecule was also located approximately 2.5–2.6 Å away from the water1. In molecule C, refinement with one water molecule resulted in a small positive *F*_*o*_*−F*_*c*_ density peak; therefore, the electron density was considered to represent a diatomic oxygen species. The refined peroxide (full occupancy) fit well into the electron density. However, the binding of peroxide was clearly different than that observed in 43JP (Figure C in S1 File). In molecule D, water1 refined closer to CuA (2.1 Å) than CuB (2.7 Å). The Cu-Cu distances were 4.3, 4.4, 4.4 and 4.5 Å for molecules A, B, C and D, respectively.

In the *deoxy* data at a 2.5-Å resolution, water was successfully refined asymmetrically closer to the CuA than CuB only in molecule D. This water was refined with full occupancy and the B-factor was 32. However, there were some minor positive *F*_*o*_*−F*_*c*_ electron density ripples at the copper site in the other molecules, which might indicate the existence of a disordered or partial water molecule. The resolution in the *deoxy* data (2.5 Å) was lower than that observed in the *met/deoxy* data (1.8 Å), which can partly explain why water was not clearly distinguishable. Nevertheless, the fully reduced copper site does not contain the water molecule.

Our conclusion is that the *deoxy* structure shows an almost completely reduced copper site or, in other words, represents the *deoxy*-form of *Ao*CO4. The Cu-Cu distances are 4.7 Å in molecules A, B and C and 4.6 Å in molecule D. The Cu-Cu distances are therefore clearly longer than those observed in *met/deoxy* structure (4.3–4.5 Å). In *met/deoxy*, one water molecule is refined between the copper ions in molecules A, B and D, but the distances from copper to water are relatively long (2.3–2.4 Å), and therefore it is not a typical *met*-form. The copper centre does not represent a fully reduced *deoxy*-form either because a clear electron density for one water molecule is obtained. The copper site has features of both *met*- and *deoxy-*forms and from this perspective, the copper centre is named as *met/deoxy*. However, some signs of *oxy*-form are obtained at least in molecule C. We believe that *met/deoxy* structure is only partly reduced, and it represents predominantly a mixture of *met-* and *deoxy*-forms.

The observed differences between the two data sets and between the molecules in the asymmetric unit are most likely due to X-ray radiation-induced reduction of the copper site. Metal ions are known to be particularly sensitive to photo-reduction [22–23]. An increased X-ray dose has also been shown to induce structural changes in the copper sites of laccases [24–25]. Interestingly, the signs of general radiation damage, such as partial disulphide bond breakage, were only observed in the *met/deoxy* structure, probably due to the higher resolution. Here, the alternative conformers of the disulphide-bond forming the Cys75 and Cys134 residues were observed. We cannot completely exclude the possibility that the triclinic crystal form originally represents a different form of copper site than that observed in the trigonal crystal form. Type-I monoclinic crystals of *Panulirus interruptus* haemocyanin have been shown to contain virtually only the *deoxy*-form, whereas type-II monoclinic crystals contain a mixture of the *deoxy-*, *oxy-* and *met-*forms [5].

### Re-investigation of published CBC enzyme structures

During the refinement of the *met/deoxy* and *deoxy* structures, the observed variations at the copper sites led us to investigate other published CBC structures. We analysed all of the available crystal structures of CBC enzymes and found that, in many cases, the interpretation of the electron density maps in the active site is truly problematic. In the following paragraphs, these examples are discussed in details.

#### 1BT1, 1BT2 and 1BT3 (catechol oxidase from *Ipomoea batatas*)

The catechol oxidase structures 1BT1 and 1BT3 at 2.7- and 2.5-Å resolutions have been suggested to represent the *met*-form of the enzyme with a Cu^II^-Cu^II^ distance of 3.0 and 2.9 Å, respectively [6]. The crystal structures are consistent with the spectroscopically characterized native form, suggesting a Cu-Cu distance of 2.9 Å and single oxygen atom bridge between two Cu^II^ ions [11]. In 1BT1 and 1BT3, one oxygen atom (as a restrained Cu-O-Cu unit) has been refined symmetrically between two copper ions with a distance of 2.0 Å (Table 1).

**Table 1:** Re-evaluation of copper sites in CBC enzyme structures.

		PDB_redo				re-refined		
enzyme	PDB code	form	Cu-Cu (Å)		Cu-O (Å)	form	Cu-Cu (Å)	Cu-O (Å)
IbCO	1BT1	*met*	3.0		2	*oxy*	A: 3.2 B: 3.2	1.9, 2.0
	1BT2	*deoxy*	4.4		2.2, 2.7	*deoxy*	4.2	-
	1BT3	*met*	2.9		2	*oxy*	3.0	1.9
ScTyr	2AHL	*deoxy*	4.2		2.4	dioxygen species	4.2	2.2, 2.5, 2.8, 2.1
	2ZMZ	*deoxy*	4.1		2.0, 2.4	dioxygen species	4.3	2.1, 2.7, 2.8, 2.0
	1WX2	*oxy*	3.5		2.0, 1.9, 2.1, 1.9	-		
VvPPO	2P3X	*met*	4.2		2.8	*met/deoxy*	4.3	2.3, 2.5 (water)
BmTyr	4J6T	*met*	A: 3.7 B: 3.9		2.1	A: *hydroperoxide* B: *met*	A: 3.7 B: 4.0	A: 2.0, 2.1, 2.9, 3.2 B: 2.1

**Fig 5:**
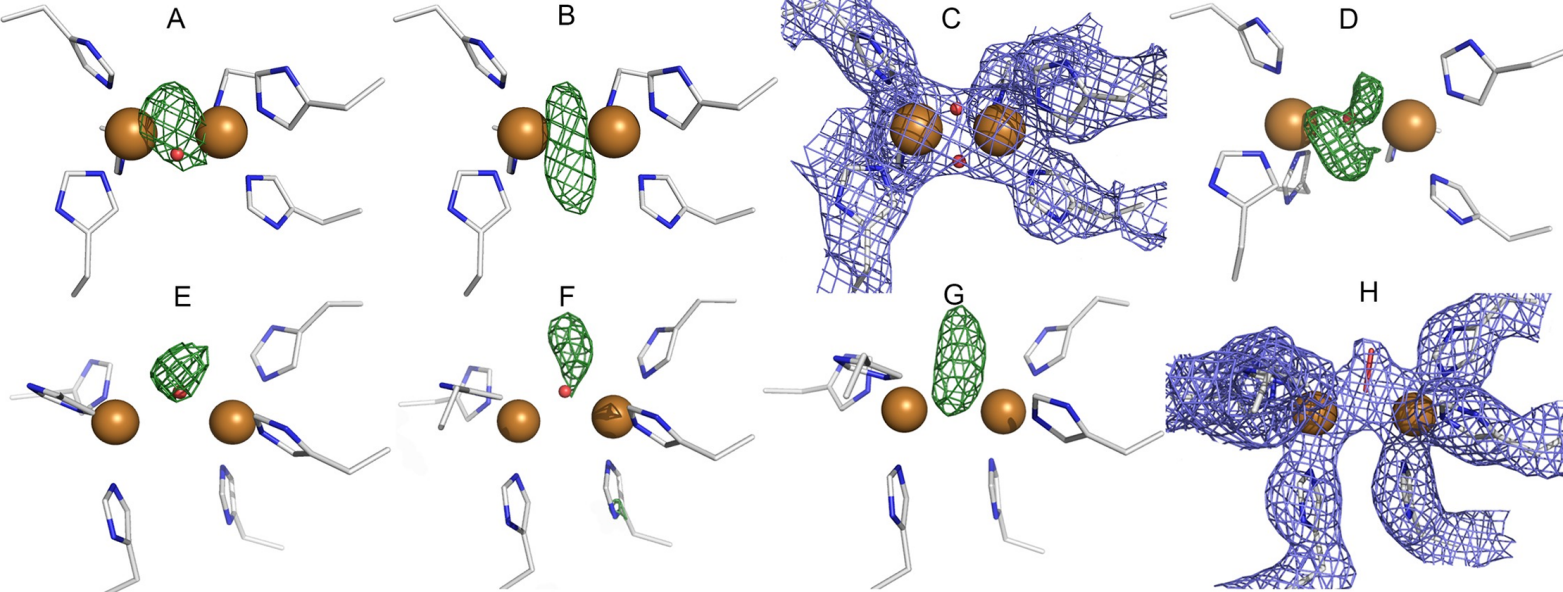
Re-refinement of publish CBC enzyme crystal structures. **A** The copper site of the deposited 1BT3 (catechol oxidase from *Ipomoea batatas*) shows a positive peak of the *F*_*o*_*−F*_*c*_ electron density (in green) in PDB_REDO-calculated maps. The distance between copper ions was 2.9 Å, and the Cu-O distances were both 2.0 Å. **B** Calculated *F*_*o*_*−F*_*c*_ difference-Fourier omit map (in green) for oxygen in 1BT3. **C** Calculated *2F*_*o*_*−F*_*c*_ Fourier map (in blue, 1σ contour level) for the copper site in the re-refined structure of 1BT3. Two oxygen atoms were refined between the copper ions. The distance between copper ions was 3.0 Å, and all the Cu-O distances were 1.9 Å. **D** The copper site of the deposited 2AHL (tyrosinase from *Streptomyces castaneoglobisporus*) shows peaks of positive *F*_*o*_*−F*_*c*_ electron density (in green) around the water in the PDB_REDO-calculated maps. **E** Copper site of deposited 2P3X with the Cu-O-Cu unit shows a peak of *F*_*o*_*−F*_*c*_ difference electron density (in green) in PDB_REDO-calculated maps. **F** Copper site of deposited 4J6T (tyrosinase from *Bacillus megaterium)* with one water molecule shows a peak of *F*_*o*_*−F*_*c*_ difference electron density (in green) in PDB_REDO-calculated maps. Molecule A is shown. **G** The calculated *F*_*o*_*−F*_*c*_ difference-Fourier omit map (in green) for water in 4J6T. **H** Calculated *2F*_*o*_*−F*_*c*_ Fourier map (in blue) for the copper site in the re-refined structure of 4J6T. Peroxide ion was refined between the copper ions. The difference maps are all shown in 3σ contour level.

In this study, the electron density maps were first checked with EDS and then calculated also through PDB_REDO. PDB_REDO optimized electron density maps showed that a positive *F*_*o*_*−F*_*c*_ density peak was observed at the copper sites of 1BT1 and 1BT3 (Fig 5A), suggesting that more than one oxygen atom could be bound. We next omitted the oxygen atom and calculated the *F*_*o*_*−F*_*c*_ electron density maps (Fig 5B), which also showed a continuous and elongated electron density for the bound species. This led us to re-refine the structure with two oxygen atoms at the copper site. The resulting *2F*_*o*_*−F*_*c*_ electron density map (Fig 5C) fit well into the model, and the residual *F*_*o*_*−F*_*c*_ electron density no longer observed. The distance between the two copper ions was increased to 3.2 Å and 3.0 Å in 1BT1 and in 1BT3, respectively. Cu-O distances of 1.9–2.0 Å were observed in 1BT1 and 1.9 Å in 1BT3. In 1BT1, the two oxygen atoms formed a bond (1.2 Å in molecule A and 1.4 Å in molecule B); however, in 1BT3, the oxygen atoms refined further away from each other (1.9 Å) and did not form a bond.

In addition to the above-mentioned structure, a *deoxy*-structure of a catechol oxidase (1BT2) with a reduced copper site Cu^I^-Cu^I^ has been solved at a 2.7-Å resolution. Here, one oxygen atom (as Cu-O-Cu unit) has been refined between the two copper ions with distances of 2.2 and 2.7 Å. However, PDB_REDO maps do not show the *2F*_*o*_*−F*_*c*_ electron density at all for the refined oxygen. When the oxygen was removed, the Cu-Cu distance was shortened to 4.2 Å. Thus, this structure represents the totally reduced copper site, and there is nothing bound in the binuclear centre.

#### 2AHL, 2ZMZ, 1WX2 (tyrosinase from *Streptomyces castaneoglobisporus*, *Sc*Tyr)

A high-resolution structure at 1.6 Å (2AHL) from a crystal soaked with hydroxyl amine has been solved. Soaking is assumed to lead to the *deoxy*-form of *Sc*Tyr, but one water is refined at the copper site. The distance between copper ions is 4.2 Å, and the distances from CuA and CuB to water are 2.4 Å. Based on the PDB_REDO electron density maps, residual positive *F*_*o*_*−F*_*c*_ electron densities were seen around the refined water between the copper ions (Fig 5D). We tried to refine a peroxide ion instead of water at this location; however, a minor *F*_*o*_*−F*_*c*_ electron density peak was seen. Due to the ambiguity of the electron density maps, it is practically impossible to draw conclusions on the bound species. We found similar observations for 2ZMZ, which is a higher resolution structure (1.37 Å) of a hydroxyl amine-treated tyrosinase crystal. We suggest that the copper site does not represent the *deoxy*-form, although the form of the copper site is unclear. The distance between copper ions is 4.2 Å, also indicating that this structure is not completely in the *deoxy*-form.

The structure of 1WX2 at a 1.8-Å resolution has been described to represent the *oxy*-form, which was achieved by soaking crystals with hydrogen peroxide [7]. A Cu-Cu distance of 3.5 Å was observed, and the distances from CuA to O1 and O2 were 2.0 and 1.9 Å, respectively, and those from CuB to O1 and O2 were 2.1 and 1.9 Å, respectively. Based on the PDB_REDO-calculated maps, only a diminutive residual positive *F*_*o*_*−F*_*c*_ density peak remained in the copper site. We concluded that the refined peroxide fits rather well into the electron density; thus, we can confirm that this structure represents the *oxy*-state of the enzyme, in agreement with the already published structure. Interestingly, a clear large residual peak of *F*_*o*_*−F*_*c*_ electron density exists in the *o*-position of Tyr98 residue of a caddie protein (Figure D in S1 File). This residue protrudes into the active site and is located approximately 4.3 Å from copper ions. Our interpretation is that this Tyr98 residue is oxygenated at the 3-position.

#### 2P3X (polyphenol oxidase from *Vitis vinifera*) at 2.2-Å resolution

The copper site was originally refined with the Cu-O-Cu unit as the *met*-form. In this structure, the Cu-Cu distance was 4.2 Å and the Cu-O distance was 2.8 Å [26]. In addition, a water molecule was located 2.9 Å away from the oxygen. However, based on the PDB_REDO electron density maps and calculated omit maps, the oxygen atom was placed incorrectly (Fig 5E). When the copper site was re-refined with a water molecule, the Cu-Cu distance was slightly increased to 4.3 Å and the distances from copper ions to water decreased to 2.3–2.5 Å. Because the Cu-Cu distance was 4.3 Å, it is likely that the copper ions are at least partly in the reduced form. In the 2P3X structure, the radiation damage is evident—i.e., the strong negative *F*_*o*_*−F*_*c*_ electron density peak is seen at disulphide bonds Cys25-Cys88 and Cys11-Cys26, suggesting that these disulphide bonds are broken. Therefore, our conclusion is that this structure predominantly represents a mixture of *met-* and *deoxy*-forms.

#### 4J6T (F197A variant of tyrosinase from *Bacillus megaterium*)

The crystal structure has been solved at a 2.4-Å resolution [27]. The copper site was refined as the *met-*form with one water molecule between the copper ions. The distance between CuA and CuB was 3.7 Å in molecule A and 3.9 Å in molecule B. The distance between CuA and water and CuB and water was 2.1 Å in both molecules. In molecule A, the PDB_REDO-calculated electron density maps showed a small positive *F*_*o*_*−F*_*c*_ peak above the water molecule (Fig 5F). Molecule B showed only a minor positive electron density. Water was therefore omitted from molecule A (Fig 5G) and the copper site was re-refined with the peroxide moiety (Fig 5H). After refining with peroxide, the *F*_*o*_*−F*_*c*_ electron density was no longer seen. Interestingly, the peroxide was refined in a similar fashion to that in the 4J3P structure of *Ao*CO4. The distance between the copper ions was 3.7 Å in molecule A with CuA-O1, CuA-O2, CuB-O1 and CuB-O2 distances of 2.1 Å, 3.2 Å, 2.0 Å and 2.9 Å, respectively.

#### 4J3P (catechol oxidase from *Aspergillus oryzae*)

We also re-refined our previous crystal structure of *Ao*CO4. In this structure, a bound dioxygen species was observed between the copper ions [20]. The published structure was refined with dioxygen with Cu-O distances of 2.0–2.6 Å. PDB_REDO-calculated electron density maps also suggested the presence of diatomic oxygen species. However, our new interpretation is that the structure should be refined with peroxide (or hydroperoxide). In principle, diatomic oxygen can be refined as dioxygen molecule O_2_ with an O-O bond distance of 1.2 Å or peroxide ion (O_2_^2-^ or OOH^-^) with an O-O bond distance of 1.4 Å. Peroxide and hydroperoxide are not distinguishable, because the hydrogens cannot be observed. No differences in the electron density maps were observed whether dioxygen or peroxide was refined. However, the coordination geometry implied that hydroperoxide might be bound.

Here reported structures 5OR3 and 5OR4 (*met/deoxy* and *deoxy)* of *Ao*CO4 were also run through PDB_REDO and optimised electron density maps were analysed. For 5OR3, the copper site in molecules A, B and D were very similar than those observed in our PHENIX refined structure. In molecule C, however, the Cu-Cu distance was increased from 4.4 to 4.5 Å and peroxide was refined slightly differently. In 5OR4 structure, the copper site had positive *F*_*o*_*−F*_*c*_ electron density in molecules A, C and D, but the copper site was totally in *deoxy*-form in molecule B. Cu-Cu distance in molecule B was increased from 4.6 to 4.7 Å, but other distances were the same. PDB_REDO uses REFMAC5 [28] from CCP4 package in structure refinement which explains minor differences at the copper site. We also observed some minor differences when 5OR3 and 5OR4 structures were refined with REFMAC5. For 5OR3 structure, the electron density in molecule C suggested that water could be refined between copper ions instead of peroxide. In other molecules, no differences between PHENIX [29–30] and REFMAC5 refined structures were seen. In case of 5OR4, REFMAC5 refinement resulted to slightly stronger positive *F*_*o*_*−F*_*c*_ peaks at the copper site of molecules A, C and D. However, these peaks were not sufficient to refine water molecules. As a summary, this supports the conclusion that 5OR3 mostly represents the *met/deoxy*-form and 5OR4 represents the *deoxy*-form.

### Different forms of coupled binuclear copper sites

**Fig 6:**
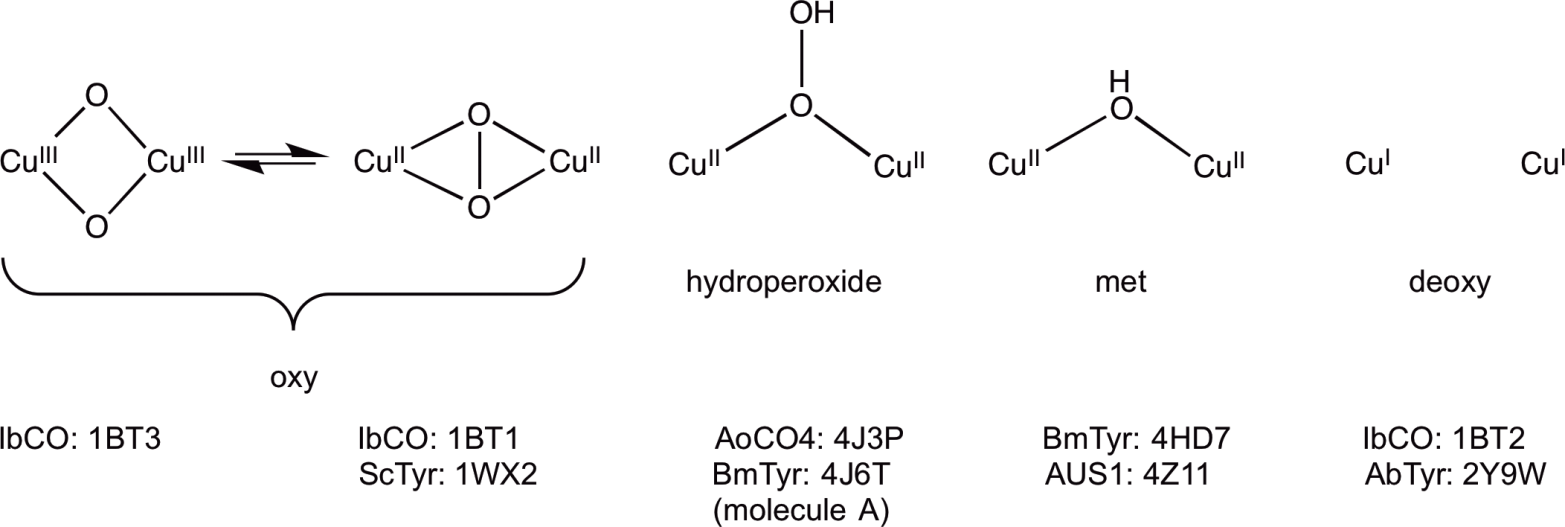
Different forms of coupled binuclear copper sites.

Based on crystal structures and spectroscopic measurements of binuclear copper enzymes, four main forms of the copper sites can be characterized, namely *oxy*, *met*, *hydroperoxide* and *deoxy* (Fig 6). Evidently, these structures represent major intermediates in the reduction pathway starting from molecular oxygen and can be used in the elucidation of the catalytic mechanism of binuclear copper enzymes.

Our conclusion is that the copper site of 1BT3 (*Ib*CO) exists in the bis-*μ*-oxo isomer of the *oxy*-form due to the short Cu-Cu distance of 3.0 Å. Based on EXAFS measurements of *Ib*CO, Cu-Cu distance of 2.9 Å for the native resting state and 3.8 Å for *oxy*-form are obtained. However, the short Cu-Cu distance of 2.7–2.9 Å has been found particularly for bis-*μ*-oxo isomer [31]. In addition, XANES spectra of *Ib*CO show that the observed pre-edge peak at 8982 eV in the resting state of the enzyme will disappear when enzyme is treated with hydrogen peroxide. The peak at 8979 eV is thought to be characteristic for Cu(II) in the side-on peroxo species and it will shift up to 8981 eV for Cu(III) [17]. Cu-complex biomimetic studies have shown that *oxy*-form exists in equilibrium between *μ-η*^2^:*η*^2^ side-on-peroxo and bis-*μ*-oxo species [32]. The two isomers differ in the Cu-Cu distance having a distance of 2.8 Å for bis-*μ*-oxo and 3.5–3.8 Å for side-on-peroxo species [11]. We therefore assume that the resting state of *Ib*CO might be a mixture of *met*- and *oxy*-forms and equilibrium can be turned towards side-on peroxo species by adding peroxide. The re-refinement of the crystal structure of 1BT3 with two oxygen atoms resulted in good-quality *2F*_*o*_*−F*_*c*_ maps, and no residual *F*_*o*_*−F*_*c*_ electron density was observed. In addition, the observed O-O distance of 1.9 Å in the re-refined structure of 1BT3 was consistent with the calculated distance [33].

In side-on-peroxo species, a peroxide coordinates the two Cu^II^ ions; however, in bis-*μ*-oxo species, the bond in peroxide is broken by two electrons from copper ions, resulting in oxidized Cu^III^ ions. The data sets of *Ib*CO crystals were collected by a RIGAKU rotating anode X-ray source; consequently, the reduction of copper ions is presumably minimal. The side-on peroxide form was found in a crystal structure of 1WX2 tyrosinase with a Cu-Cu distance of 3.5 Å. Our crystallographic analysis also suggested that the structure of *Ib*CO 1BT1 could be predominantly in the *μ-η*^2^:*η*^2^ side-on-peroxo form or probably a mixture of the two *oxy*-isomers. The Cu-Cu distance of 3.2 Å was detected in our re-refined structure of 1BT1. By contrast, the coordination of the peroxide ion is completely different in 4J3P. Based on our analysis, the crystal structure of 4J6T could have a similar species bound in the copper site of molecule A.

The *met*-form of the binuclear copper site has been observed in the structures of the *Bm*Tyr mutant (4HD7) [27] at a 2.1-Å resolution and aurone synthase from *Coreopsis grandiflora* (4Z11) at a 2.5-Å resolution [34]. In the 4HD7 structure, the distance between the copper ions is 4.0 Å in molecules A and B, and a water molecule is refined between them. The water is located slightly closer to CuA than CuB in both molecules. The distance between CuA to water and CuB to water are 2.0–2.1 Å and 2.2–2.4 Å, respectively. In the 4Z11 structure, the average Cu-Cu distance is 4.1 Å. This *met*-form is refined with a water molecule between the copper ions, and the distances are in the range of 2.1–2.3 Å.

The re-refinement of structure 1BT2 showed that the copper site is fully reduced and nothing is bound between the copper ions. Our observed *deoxy* structure also showed the reduced copper site with Cu-Cu distances of 4.6–4.7 Å. Additionally, a structure 2Y9W of tyrosinase from *Agaricus bisporus* at 2.3-Å resolution represents the *deoxy*-form [35], but a water molecule is lying between the copper ions. The Cu-Cu distances in molecules A and B are 4.5 and 4.4 Å, respectively. The distances from CuA and CuB to water are 3.0 and 2.6 Å in molecule A and 2.7 and 2.4 Å in molecule B, respectively. Thus, it is likely that the bound species is presumably water, not a bridged hydroxide ion as seen in *met*-form.

## Conclusions

Two crystal structures of catechol oxidase from *Aspergillus oryzae*, classified as *met/deoxy* and *deoxy*, were solved at resolutions 1.8 and 2.5 Å. In the *met/deoxy* structure, the copper sites of the A, B and D molecules were refined with one water molecule between CuA and CuB. In molecule C, a peroxide ion was refined between the copper ions. In the *deoxy*-structure, nothing was bound between the copper ions in molecules A, B and D. In molecule C only, a water molecule was refined closer to CuA than CuB. Therefore, the *deoxy* structure was concluded to represent almost the completely reduced copper site, but the *met/deoxy* structure was only partly reduced and represented a mixture of *met-* and *deoxy*-forms. The observed differences between the two data sets and between the molecules in the asymmetric unit were thought to be due to the X-ray radiation-induced reduction of the copper site.

Synchrotron X-rays rapidly reduce the copper ions, contributing to the reduction of oxygen species. This may result in a mixture of different forms, which must be considered when refining and interpreting the crystal structures of binuclear copper enzymes. All of the CBC enzyme structures deposited to the PDB were evaluated and re-analysed. We suggest that the copper site of *Ib*CO in structures 1BT1 and 1BT3 may exist in an *oxy*-form. The *Sc*Tyr structures of 2AHL and 2ZMZ were not found to be, at least not completely, in the *deoxy*-form. The copper site in 2P3X of *Vv*PPO was concluded to represent a mixture of *met-* and *deoxy*-forms. In addition, a putative *hydroperoxide*-form was suggested to exist in 4J3P and in molecule A of 4J6T.

X-ray radiation-induced changes are not the only problem during crystallographic refinement of metal centres. Metal-ligand bond lengths and angles can be restrained tightly, loosely or not at all during the refinement process. Unfortunately, there is no generally accepted uniform strategy for the refinement of metal sites [36]. Furthermore, there is no rule regarding how to refine mono/diatomic oxygen species into the binuclear copper site. Because hydrogens cannot be observed, monoatomic oxygen can be refined as an oxygen atom (O^2-^ or OH^-^) or as a water molecule. Additionally, diatomic species can be refined as a bound dioxygen, peroxide (O_2_^2-^ or OOH^-^), or with two closely associated oxygen atoms with a distance of 1.9 Å. Again, various bond length and angle restraints for the bound oxygen species can be used. Therefore, the refinement of metal centres, at least partly, depends on the interpretation of the crystallographer and it seems that it is impossible to obtain the absolute form of a copper site by X-ray crystallography. Neutron crystallography would be the only experimental method to obtain hydrogens and it could be used to study the full reaction pathway.

## Methods

### Purification and crystallization

Catechol oxidase from *Aspergillus oryzae* (*Ao*CO4) was expressed in *Trichoderma reesei* and was produced in a fermenter as previously described by Gasparetti et al. [12] *Ao*CO4 was purified in two steps—i.e., anion exchange chromatography, followed by size exclusion chromatography. The concentrated culture supernatant was first desalted with a ready-to-use PD-10 column containing SephadexTM G-25 (GE Healthcare) in Tris-HCl buffer (20 mM, pH 7.2). The desalted sample was applied to a Resource Q (6ml) column (Pharmacia Biotech) in Tris-HCl buffer (20 mM, pH 7.2). Purification was performed with ÄKTA purifier (GE Healthcare Life Sciences). Bound proteins were eluted with a linear sodium chloride gradient (0–200 mM in 20 column volumes) in the equilibrium buffer. Active fractions were pooled, desalted, and concentrated with Vivaspin (5 kDa MWCO) (GE Healthcare Life Sciences) and were subsequently applied to a column Superdex 75 HR 10/30 (24 ml) (Amersham Biosciences) in Tris-HCl buffer (20 mM, pH 7.2). Chromatographs were evaluated using UNICORN 5.01-software. Active fractions were again pooled and concentrated. Purified *Ao*CO4 was examined by SDS-PAGE with silver staining (Pharmacia LKB, Phast System).

Crystallization was performed using the hanging-drop vapour diffusion method in Linbro-style 24-well plates (Greiner CELLSTAR) at room temperature. AoCO4 was crystallized by a reservoir solution (10% PEG 20,000, 6% ethylene glycol and 0.1 M sodium citrate at pH 4.0). The crystallization drop contained *Ao*CO4 (2 μl, 6–8 mg/ml) solution and reservoir solution (2 μl). The hanging drop was equilibrated against reservoir solution (500 μl). Long stick-like crystals were observed after 24 hours, and the crystals grew to their final size in approximately three days.

### Data collection and structure determination

Crystals were harvested and plunged into liquid nitrogen. Ethylene glycol (30%) served as a cryo protectant. Two different data sets (*met/deoxy* and *deoxy*) from two crystals were collected. The diffraction data of the *met/deoxy* crystal were collected at Diamond Light Source (DLS), Oxfordshire, England at MX beamline i02. The diffraction data of the *deoxy* crystal were collected at the European Synchrotron Radiation Facility (ESRF), Grenoble, beamline ID23-2. Both data sets were processed with the XDS software package [37]. Data collection statistics are shown in Table 2.

**Table 2:** Data collection and structure refinement statistics for *Ao*CO4 crystals.

	*met/deoxy* (5OR3)	*Deoxy* (5OR4)
Beamline	Diamond i02	ESRF ID23-2
Wavelength (Å)	0.976250	0.87260
Resolution (Å)	1.8	2.5
Space group	P1	P1
Unit cell: a (Å)	60.7	60.8
b	81.5	81.9
c	82.3	82.6
α (°)	87.2	82.6
β	89.2	89.1
γ	73.9	73.9
Observed reflections	485,321 (73,791)	94,332 (12,034)
Unique reflections	136,838 (21,443)	53,530 (7,542)
Completeness	94.2 (97.1)	92.2 (80.5)
*R*_*obs*_ (%)	11.4 (88.2)	14.3 (54.0)
*I/σ(I)* (%)	7.37 (1.26)	6.19 (1.78)
*CC1/2*	60.1	47.0
No. of reflections	136,809	53,469
Molecules in ASU	4	4
*Rwork/Rfree* (%)	17.6/20.7	20.3/26.1
**No. of atoms**	13,291	12,320
Amino acids	11,645	11,517
Copper ions	8	8
Ligands	404	340
Waters	1,242	463
**B-factors (Å)**	30.3	28.8
Amino acids	28.9	28.8
Copper ions	30.3	33.7
Ligands	50.0	46.4
Waters	36.1	27.1
**RMS deviations**		
Bond length (Å)	0.011	0.003
Bond angles (°)	1.07	0.70
DPI (Å)	0.117	0.868

The crystal structures were solved by the molecular replacement method, and a structure of full-length *Ao*CO4 (PDB code 4J3P) served as a template. Phaser [38] software from the CCP4 suite, version 6.5 [39], was used in molecular replacement. Coot [40] software was used for model building, and the structures were refined with phenix.refine, version 1.11.1–2575 [29–30]. Anomalous difference Fourier maps for *met/deoxy* and *deoxy* data were calculated and used for examining the Cu positions (Figure E in S1 File). For *deoxy* data, the anomalous signal was too weak and not clearly detectable. Explicit riding hydrogen atoms were refined in the *met/deoxy* structure. Table 2 presents the statistics of structure refinement. Copper-histidine restraints with an ideal distance of 2.02 Å and variance of 0.1 Å were used in the refinement. No links between copper ions and oxygen moieties were used to avoid bias in the copper site structure. Matthews_coef, a program in the CCP4 package, determined the solvent content of crystals. The solvent content for the *met/deoxy* structure was approximately 44% (the Matthews coefficient was 2.2 Å^3^Da^-1^) and 45% for the *deoxy* structure (the Matthews coefficient was 2.3 Å^3^Da^-1^). The space group was verified to be P1 using Zanuda software from CCP4 package [41]. The final refined structures were also run through the online Diffraction Precision Index (DPI) server [42]. Atomic coordinates and structure factors have been deposited in the Protein Data Bank under accession codes 5OR3 and 5OR4.

### Dynamic light scattering

Oligomerization and homogeneity of *Ao*CO4 was studied by dynamic light scattering. Measurements were performed using DynaPro99 dynamic light scattering system (Wyatt Technology Corp.) with temperature-controlled micro sampler. *Ao*CO4 was filtered and scanned 20 times per measurement.

### Re-evaluation of CBC structures

The structure factors and coordinate files were downloaded in Coot software through PDB_REDO interface, which automatically downloads the optimized models and electron density maps. All available CBC protein structures were inspected, and the copper sites of the following structures were then further re-refined: 1BT1, 1BT3, 2AHL, 2P3X, 2ZMZ and 4J6T. Initially, water molecules or oxygen species between coppers were totally omitted, and new electron density maps were calculated. REFMAC5 [28] from the CCP4 [39] software package was used in refinement. Based on the calculated omit electron density maps, some of the structures were re-refined. No links between copper ions and oxygen moieties were used in the refinement.

## Supporting information

S1 File (Figure A) Citrate ion between two homodimers. (Figure B) The dynamic light scattering diagram for *Aspergillus oryzae* catechol oxidase. (Figure C) The copper sites of superimposed 4J3P and molecule C of *met/deoxy* structure. (Figure D) The copper site of *Streptomyces castaneoglobisporus* tyrosinase (1WX2). (Figure E) The anomalous map for copper ions in *met/deoxy* (5OR3) structure of catechol oxidase from *Aspergillus oryzae*.
(DOCX)
