# Supplementary material for: A new crystal form of Aspergillus oryzae catechol oxidase and evaluation of copper site structures in coupled binuclear copper enzymes
Source: PLoS One. 2018 May 1;13(5):e0196691. doi: 10.1371/journal.pone.0196691 (PMC5929527; doi:10.1371/journal.pone.0196691)
Supplement: S1 File — (Figure A) Citrate ion between two homodimers. (Figure B) The dynamic light scattering diagram for Aspergillus oryzae catechol oxidase. (Figure C) The copper sites of superimposed 4J3P and molecule C of met/deoxy structure. (Figure D) The copper site of Streptomyces castaneoglobisporus tyrosinase (1WX2). (Figure E) The anomalous map for copper ions in met/deoxy (5OR3) structure of catechol oxidase from Aspergillus oryzae. (DOCX) [file pone.0196691.s001.docx]

L. Penttinen et al. A new crystal form of *Aspergillus oryzae* catechol oxidase and evaluation of copper site structures in coupled binuclear copper enzymes

Supporting information

Leena Penttinen^1^, Chiara Rutanen^1^, Markku Saloheimo^2^, Kristiina Kruus^2^, Juha Rouvinen^1^, Nina Hakulinen*^1^

^1^ Department of Chemistry, University of Eastern Finland Joensuu Campus, Joensuu, Finland

^2^ VTT Technical Research Center of Finland Ltd., Espoo, Finland

* Corresponding author

E-mail: [nina.hakulinen@uef.fi](mailto:nina.hakulinen@uef.fi) (NH)

Supplementary figures


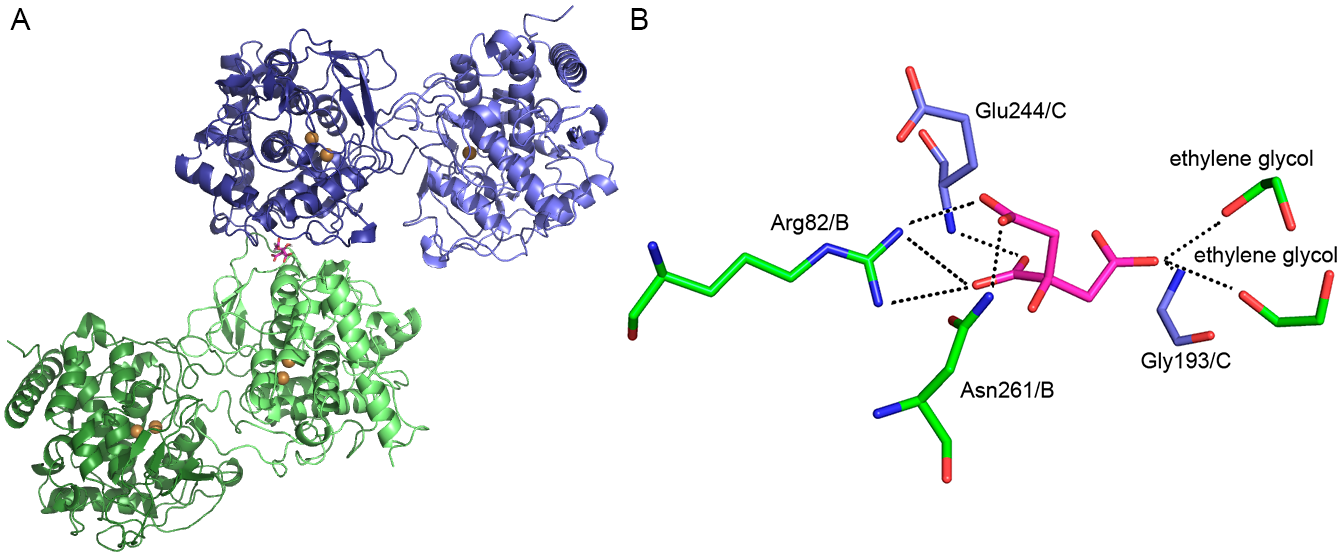


**Figure A.** **Citrate ion between two homodimers.** A) Asymmetric unit of new crystal form of *Aspergillus oryzae* catechol oxidase consists of two homodimers (shown in green and blue). Copper ions are shown as orange spheres. B) Citrate ion (shown in magenta) forms several polar contacts between the homodimers.


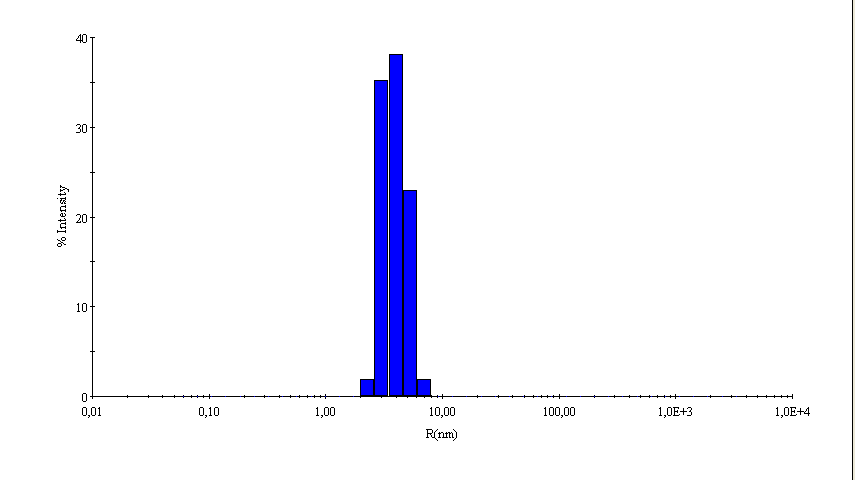


**Figure B.** **The dynamic light scattering diagram for *Ao*CO4.** A monomodal distribution with a mean size 4.0 nm was obtained.


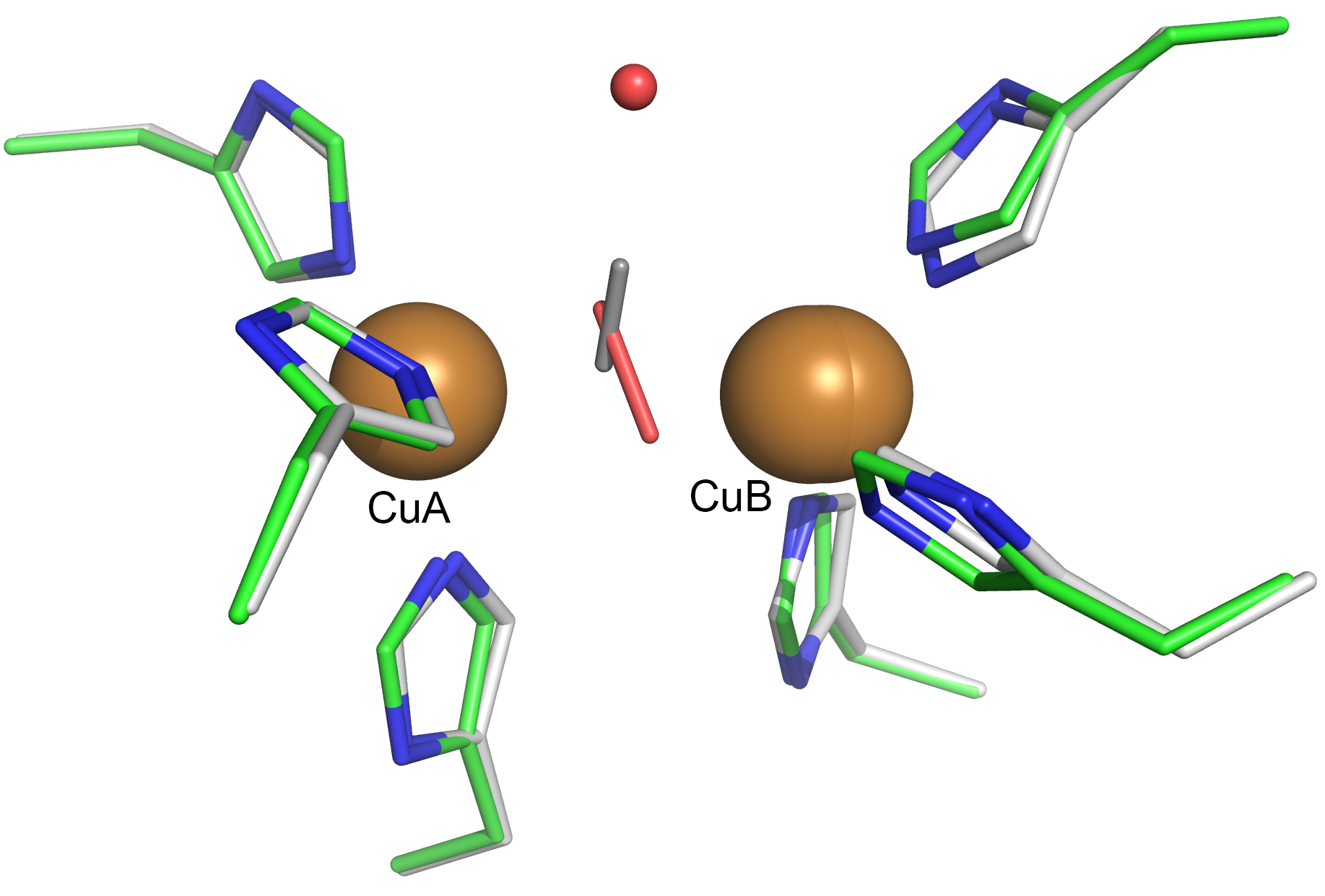


**Figure C**. **The copper sites of superimposed 4J3P and molecule C of *met/deoxy* structure.** 4J3P is represented in gray and molecule C of *met/deoxy* structure is shown in green. The peroxide is shown as a red stick and nearby water is show as red sphere.


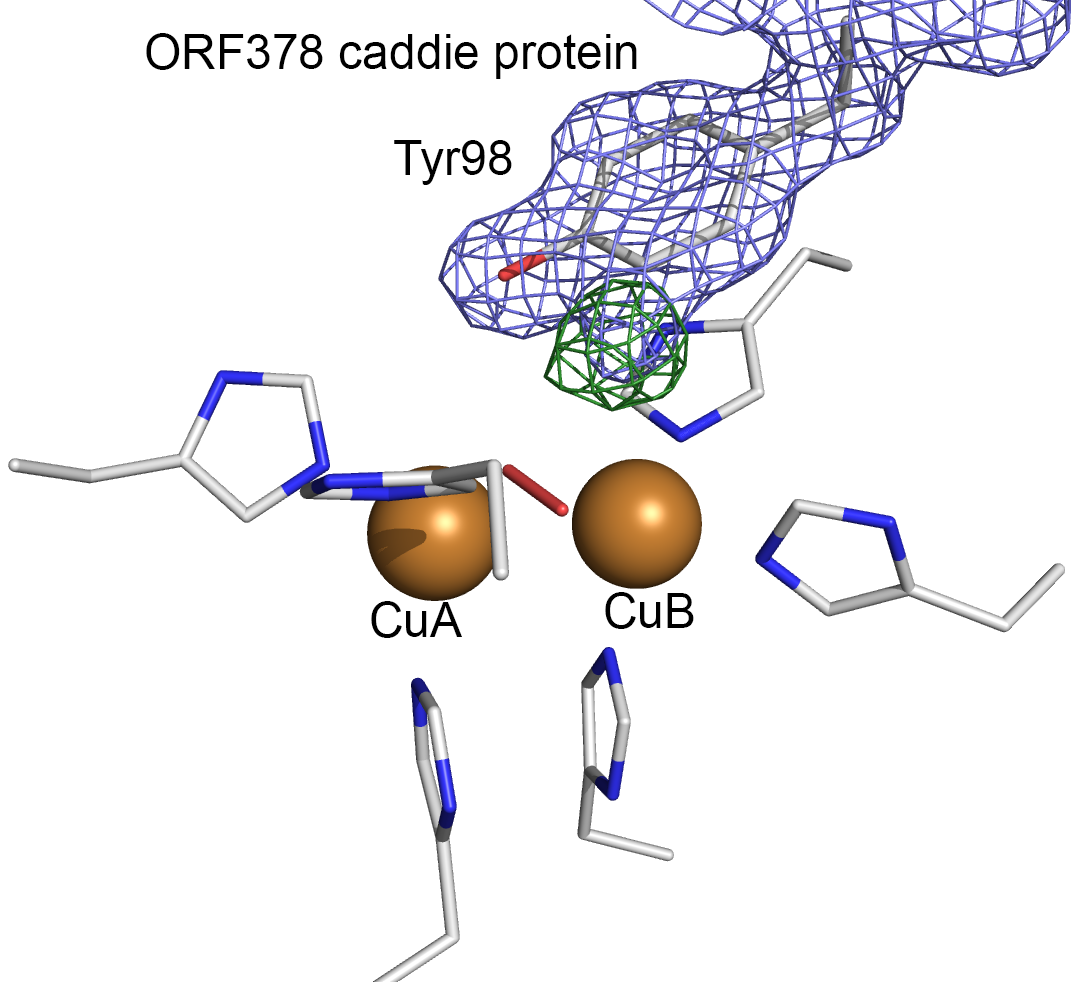


**Figure D**. **The copper site of *Streptomyces castaneoglobisporus* tyrosinase (1WX2).** The electron density map inTyr98 of caddie protein ORF378. The *F_o_ – F_c_* difference map is shown in green at 3σ contour level. The *2F_o_ – F_c_* Fourier map for Tyr98 is represented in blue at 1σ contour level.


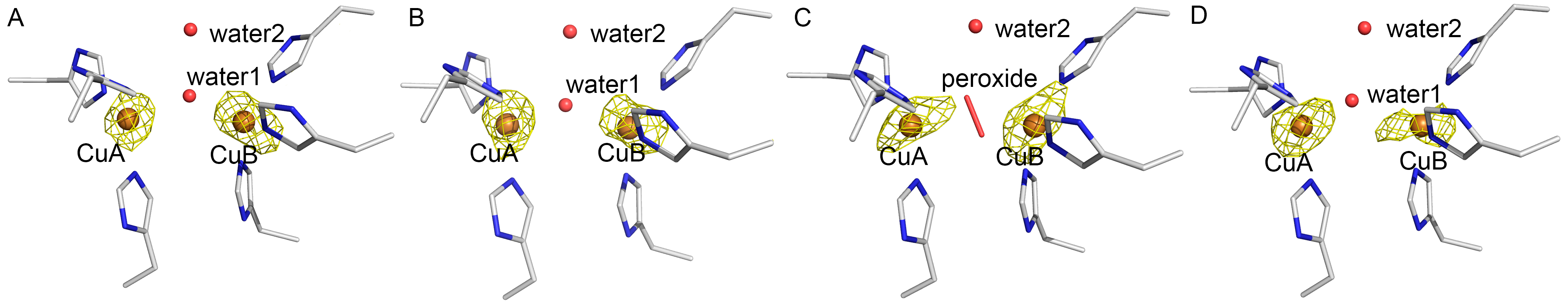


**Figure E. The anomalous map for copper ions in *met/deoxy* (5OR3) structure of catechol oxidase from *Aspergillus oryzae*.** **A,** **B**, **C** and **D** correspond molecules A, B, C and D. The anomalous map is shown in yellow at 4σ contour level.
